# A Novel Approach to Left Ventricular Filling Pressure Assessment: The Role of Hemodynamic Forces Analysis

**DOI:** 10.3389/fcvm.2021.704909

**Published:** 2021-09-08

**Authors:** Lorenzo Airale, Fabrizio Vallelonga, Tommaso Forni, Dario Leone, Corrado Magnino, Eleonora Avenatti, Andrea Iannaccone, Anna Astarita, Giulia Mingrone, Marco Cesareo, Carlo Giordana, Pierluigi Omedè, Claudio Moretti, Franco Veglio, Gianni Pedrizzetti, Alberto Milan

**Affiliations:** ^1^Internal Medicine and Hypertension Division, Department of Medical Sciences, Azienda Ospedaliera Universitaria (AOU) “Città della Salute e della Scienza” Hospital, University of Turin, Turin, Italy; ^2^Hemodynamic Laboratory, Department of Medical Sciences, Azienda Ospedaliera Universitaria (AOU) “Città della Salute e della Scienza” Hospital, University of Turin, Turin, Italy; ^3^Department of Engineering and Architecture, University of Trieste, Trieste, Italy

**Keywords:** echocardiography, hemodynamic forces, right heart catheterization, left ventricular filling pressure, diastolic function

## Abstract

**Background:** Diastolic function in patients with heart failure is usually impaired, resulting in increased left ventricular (LV) filling pressures, whose gold standard assessment is right heart catheterization (RHC). Hemodynamic force (HDF) analysis is a novel echocardiographic tool, providing an original approach to cardiac function assessment through the speckle-tracking technology. The aim of our study was to evaluate the use of HDFs, both alone and included in a new predictive model, as a potential novel diagnostic tool of the diastolic function.

**Methods:** HDF analysis was retrospectively performed in 67 patients enrolled in the “Right1 study.” All patients underwent RHC and echocardiography up to 2 h apart. Increased LV filling pressure (ILFP) was defined as pulmonary capillary wedge pressure (PCWP) ≥ 15 mmHg.

**Results:** Out of 67 patients, 33 (49.2%) showed ILFP at RHC. Diastolic longitudinal force (DLF), the mean amplitude of longitudinal forces during diastole, was associated with the presence of ILFP (OR = 0.84 [0.70; 0.99], *p* = 0.046). The PCWP prediction score we built including DLF, ejection fraction, left atrial enlargement, and e' septal showed an AUC of 0.83 [0.76–0.89], with an optimal internal validation. When applied to our population, the score showed a sensitivity of 72.7% and a specificity of 85.3%, which became 66.7 and 94.4%, respectively, when applied to patients classified with “indeterminate diastolic function” according to the current recommendations.

**Conclusion:** HDF analysis could be an additional useful tool in diastolic function assessment. A scoring system including HDFs might improve echocardiographic accuracy in estimating LV filling pressures. Further carefully designed studies could be useful to clarify the additional value of this new technology.

## Graphical Abstract

Risk variation of presenting increased left ventricular filling pressure (upper graph) and PCWP variation (lower graph), according to the proposed scoring system. EF, ejection fraction; DLF, diastolic longitudinal force; LAe, left atrial enlargement; ILFP, increased left ventricular filling pressure; NLFP, normal left ventricular filling pressure; PCWP, postcapillary wedge pressure.
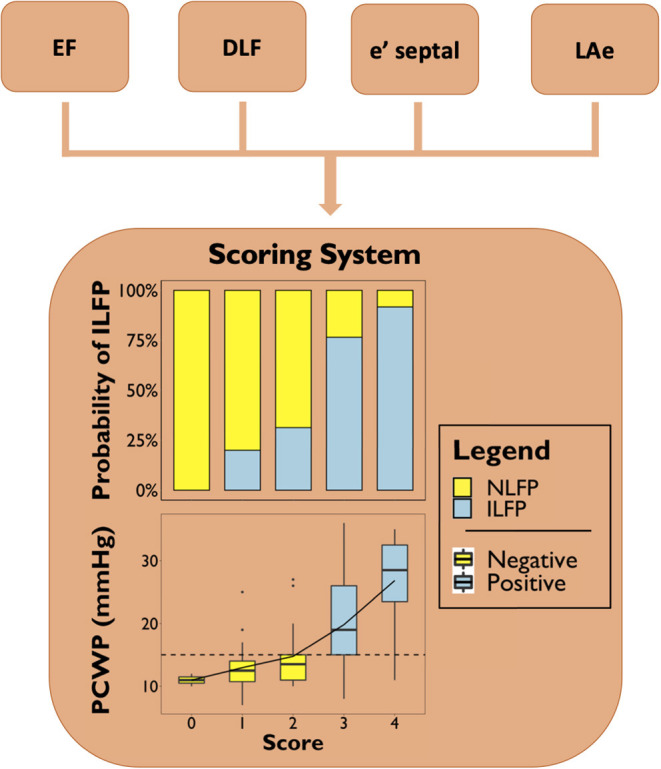

## Introduction

Heart failure involves up to 10% of the population over 75 years old; 33% of males and 28% of females aged more than 55 will present at least one episode of heart failure in their life, making heart failure one of the main causes of hospitalization in subjects over 65 years old (1). Patients affected by heart failure typically show a certain degree of left ventricle (LV) diastolic dysfunction. This feature leads to increased LV filling pressure, resulting in the postcapillary pattern of pulmonary hypertension (PH), defined by an increased pulmonary capillary wedge pressure (PCWP > 15 mmHg) measured during right heart catheterization (RHC) (2, 3).

Transthoracic echocardiography proved to be more accurate than clinical evaluation (including physical examination, chest x-ray findings, and natriuretic peptide levels) in PCWP estimation (4), becoming the routinely non-invasive diagnostic tool dedicated to the evaluation of diastolic function (5). However, several parameters and a complex flowchart are needed for this purpose (6).

Recently, many studies have introduced ventricular blood flow analysis as an innovative method to assess cardiac function (7–13). Blood motion within the LV is characterized by the development of vortices, involved in the preservation of blood kinetic energy during the diastolic phase and, consequently, in the decrease of cardiac work during systolic ejection (14, 15). However, as long as flow analysis techniques have depended on the administration of contrast agents or on the use of MRI, their spread in clinical practice has been limited. In recent years, a mathematical model, based on first principles of fluid dynamics, was able to estimate HDFs through the knowledge of LV geometry, endocardial tissue movement, and areas of the aortic and mitral orifices, without knowing blood velocities inside the LV (13). This has been possible because blood flow pattern and LV wall motion are so closely linked, that an appropriate knowledge of tissue motion (by speckle tracking analysis indeed) makes the estimation of the flow forces produced inside the cardiac chambers possible (8). Thanks to this model, HDF analysis might become a novel and more widely applicable method in clinical practice through conventional echocardiography.

To date, echocardiographic flow analysis has always been studied in relation to the systolic pattern of the cardiac cycle, particularly in patients with dilated cardiomyopathy. Hemodynamic forces (HDFs) were able to properly predict the response to cardiac resynchronization therapy (10, 11), which is itself associated with an improvement in the diastolic function (16–18). However, no studies are available about the direct relationship between HDFs and diastolic function.

The aim of this retrospective pilot study was to evaluate, in a population of patients who underwent RHC, the HDF analysis as a potential novel diagnostic tool of diastolic dysfunction, both as a single entity and included within a new predictive model, considering other conventional echocardiographic parameters.

## Methods

The Right1 Study was a prospective study, whose enrollment took place between July 2011 and November 2013, involving patients referred to the Division of Cardiology of the University of Turin with a specialistic indication for RHC (19), mainly a suspected pulmonary hypertension. It involved 190 patients without ongoing infusions of hemodynamically active drugs, known pulmonary stenosis, or ventilator support.

In the present study, we retrospectively analyzed clinical and instrumental data of the patients enrolled in the Right1 Study, excluding those with atrial fibrillation or pacing devices. Adequate echocardiographic windows were required to be analyzed by a dedicated software: visualization of endocardial borders throughout the whole cardiac cycle and proper image contrast between endocardial borders and blood.

The Right1 Study was approved by our local ethic committee (Comitato Etico Interaziendale A.O.U. Città della Salute e della Scienza di Torino – A.O. Ordine Mauriziano), and all patients provided written informed consent before enrollment, even authorizing the retrospective use of the records for scientific purposes.

### Right Heart Catheterization

RHC was performed through femoral or jugular access. Pulmonary capillary wedge pressure (PCWP) was acquired with the zero-reference level always set at the midthoracic level. All measurements were made at end expiration. Hemodynamic values were interpreted according to an international consensus (18). Physicians performing the RHC were blinded to the results of the transthoracic echocardiography.

### Echocardiography

Transthoracic echocardiography was performed before RHC, within 2 h of the examination, by an experienced operator, with a commercially available machine (IE33, Philips, The Netherlands) equipped with a S5 probe for two-dimensional and Doppler acquisition. All echocardiographic measurements were performed following the current international recommendations, while the patient was in left lateral decubitus (20).

Left ventricular hypertrophy (LVH) was defined as a left ventricular mass (LVM) normalized for BSA (LVMi) > 95 g/m^2^ in women or > 115 g/m^2^ in men (20). According to the current recommendation (6), PCWP was considered abnormal if >15 mmHg (increased left ventricular filling pressure, ILFP) and left atrial enlargement (LAe) if left atrial volume normalized for BSA (LAVi) > 34 ml/m^2^; septal TDI-E-wave (e' septal) and lateral TDI-E-wave (e' lateral) were considered pathological when <7 cm/s and <10 cm/s, respectively, transmitral PW-E-wave/mean TDI-E-wave (E/A) when >14, and maximum tricuspid regurgitation velocity (TRv) when >2.8 m/s.

BSA was calculated using the Dubois and Dubois formula (21):

$$\begin{array}{l} BSA\left[m^{2}\right]=0.20247\;*\;weight{\left[kg\right]}^{0.425}\;*\;height{\left[m\right]}^{0.725} \end{array}$$

### HDFs Evaluation

HDFs were obtained by off-line analysis of echocardiographic DICOM files with a dedicated software (QStrain Echo Prototype v.1.3, Medis Medical Imaging, Leiden, The Netherlands). Speckle-tracking analysis of LV was performed in the three routinely acquired apical scans: four-chamber, two-chamber, and three-chamber views. HDFs can be detected through endocardial velocities, LV geometry, and aortic and mitral orifices areas, obtained after measuring the internal diameter of the valve anulus in parasternal long axis-view (8, 22). In particular, the force vector is given at every instant during the heart cycle by its definition, which is either the integral of blood flow velocity inside the ventricle volume *V*_*LV*_ (first integral in the formula below)

$$\begin{array}{l} F\;=\rho \int_{V_{LV}}\left(\frac{\partial v}{\partial t}+v\cdot \nabla v\right)\;dV\\ \;=\rho \int_{S_{LV}}\left[x\left(\frac{\partial v}{\partial t}\cdot n\right)+v\left(v\cdot n\right)\right]\;dS\; \end{array}$$

or the integral on the surface *S*_*LV*_ surrounding of the same volume (second integral in the formula below).

The present study used the second formulation. In that computation, the velocity values on the tissue part of the surface *S*_*LV*_ are given directly from speckle tracking. The average velocity of blood on the open part of the boundary *S*_*LV*_ (e.g., the mitral area, during diastole) is estimated by mass conservation (in diastole, the relative velocity times the mitral area is equal to the LV volume rate). The longitudinal component of the HDF is then taken as the component of the vector that is parallel to the direction of the LV axis. In order to make patients with different LV sizes comparable, the instantaneous value of HDFs has been normalized by the corresponding value of LV volume. It was then divided by blood density and gravity acceleration, obtaining a dimensionless value that corresponded to the force expressed as a percentage of gravity acceleration (22).

**Figure 1:**
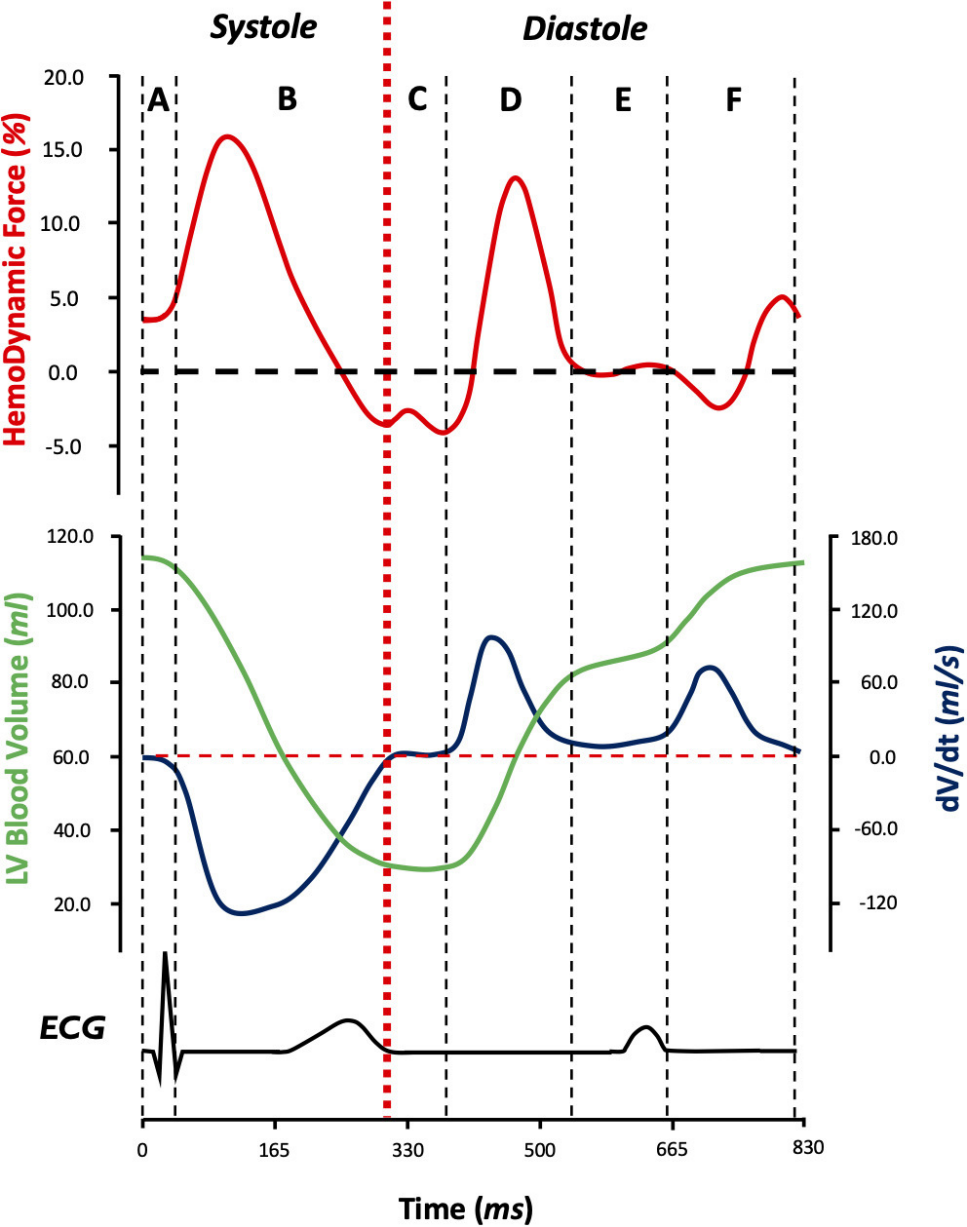
Longitudinal hemodynamic force pattern (red) in relation to left ventricular blood volume (green), left ventricular blood volume change velocity (blue), and electrical activity during the cardiac cycle (black) during the six phases of the cardiac cycle **(A–F)**: isovolumic contraction **(A)**, systolic ejection **(B)**, isovolumic relaxation **(C)**, early diastolic filling **(D)**, diastasis **(E)**, and late diastolic filling **(F)**. By convention, when the HDF vector is directed from the apex to base of LV (when apical pressure is higher than basal pressure), it is considered to be positive (above the zero line) and when the HDF vector is directed from the base to apex (basal pressure higher than apical pressure), it is considered to be negative (below the zero line). DLF is described by root mean square of the diastolic segment of longitudinal hemodynamic forces. ECG, electrocardiography; LV, left ventricle; DLF, diastolic longitudinal force. dV/dt, derivative of volume as a function of time (left ventricular blood volume change velocity).

Figure 1 displays a typical time profile of HDF. In particular, we took into account diastolic longitudinal force (DLF) as a parameter describing the diastolic behavior of HDF. DLF is defined as the mean amplitude, expressed as root mean square, of the longitudinal force throughout the diastolic part of the cardiac cycle (Figure 1A).

### Statistical Analysis

Statistical analysis was performed by using a dedicated software (R: A Language and Environment for Statistical Computing, v4.0.0 for Mac OSX, R Core Team., Vienna, Austria). The normal distribution of variables was verified by graphical evaluation (histogram and Q-Q graph) and Shapiro–Wilk test. Data were presented as “mean ± standard deviation” or “median [interquartile range]” and as “observations (percentage frequency)” as appropriate. Differences between groups were analyzed by *t*-test or Mann–Whitney test for continuous variables and Yates' χ^2^ test or Fisher exact test for categorical ones. Univariate logistic regression analysis was performed for all clinical variables, and a multivariate penalized regression was performed for selecting variables to be included in the multivariate model: betas of regression were shrunken toward zero and variables whose beta reached zero were excluded from subsequent analyses. The scoring system points were assigned by rounding betas of the multivariate penalized model (23) to the unit, and internal validation was assessed by bootstrap. Multicollinearity among variables included was excluded through variance inflation factor analysis. Sensibility and specificity between different methods were performed through McNemar test among patients with increased and normal LV filling pressure, respectively (24). The additional contribution of DLF in predicting the outcome was performed by net reclassification index (NRI) (25).

A *p* < 0.05 for two-tailed tests was considered significant in all statistical analysis.

## Results

Out of 190 enrolled patients, 148 met inclusion criteria. Among these, 81 patients were excluded (31 patients due to poor quality of ECG gating or presence of extrasystoles during acquisitions and 50 patients due to inadequate image quality to perform speckle-tracking analysis). Thus, the study population was composed of 67 patients, whose demographic and echocardiographic features are resumed in Table 1. The included patients did not significantly differ from the excluded ones, except for LVMi and LVH rate, as shown in Supplementary Table 1.

**Table 1:** Demographic and echocardiographic characteristics of the study population.

	**NLFP**	**ILFP**	***p*-value**
	***n* = 34**	***n* = 33**	
**Demographic characteristics**			
Age (years)	60.3 ± 12.4	64.1 ± 13.7	0.242
Sex (Male) [*n* (%)]	18 (52.9)	23 (69.7)	0.248
Weight (kg)	74.0 ± 14.4	69.5 ± 14.8	0.211
Height (cm)	166.0 ± 10.2	167.0 ± 8.1	0.506
BMI (kg/m^2^)	27.0 ± 4.91	24.8 ± 4.58	0.065
BSA (m^2^)	1.81 ± 0.20	1.77 ± 0.20	0.447
SBP (mmHg)	133.0 ± 13.9	131.0 ± 26.8	0.785
DBP (mmHg)	74.3 ± 11.7	72.4 ± 13.4	0.647
HR (bpm)	68.3 ± 11.2	65.3 ± 9.8	0.240
**Conventional echocardiographic parameters**			
EF (%)	58.6 [53.0; 63.6]	52.2 [30.7; 60.8]	**0.012**
GLS (%)	−19.4 [−21.5; −17.0]	−16.0 [−21.2; −9.4]	**0.031**
LVMi (g/m^2^)	140 ± 46.5	161 ± 40.8	0.092
RWT	0.42 ± 0.13	0.36 ± 0.11	0.040
LVH [*n* (%)]	30 (88.2)	29 (90.6)	0.967
LAVi (ml/m^2^)	34.7 [29.0; 42.8]	54.2 [40.5; 66.1]	** <0.001**
LAe [*n* (%)]	18 (52.9)	28 (84.8)	**0.010**
E (cm/s)	65.5 [56.3; 76.3]	78.0 [62.0; 87.0]	0.087
E/A	0.9 [0.7; 1.1]	1.2 [0.8; 1.8]	**0.031**
e' septal (cm/s)	6.2 [4.7; 8.6]	4.4 [3.5; 6.3]	**0.017**
e' lateral (cm/s)	8.7 ± 3.6	7.7 ± 3.6	0.289
E/e' average	9.1 [7.0; 10.7]	10.8 [9.1; 15.5]	**0.010**
TRv (m/s)	2.8 ± 0.5	2.8 ± 0.6	0.582
EDVi (ml/m^2^)	60.1 ± 17.9	84.5 ± 39.3	**0.002**
**Hemodynamic forces**			
DLF (%)	6.9 ± 3.6	5.2 ± 3.1	**0.034**
**Echo-estimated LV filling pressure**			
Normal [*n* (%)]	12 (52.2)	11 (47.8)	0.865
Indeterminate [*n* (%)]	18 (75.0)	6 (25.0)	**0.003**
Increased [*n* (%)]	4 (20.0)	16 (80.0)	**0.001**
**Right heart catheterization**			
PCWP (mmHg)	12.00 [10.00; 13.00]	25.00 [17.00; 29.00]	** <0.001**
mPAP (mmHg)	25.15 ± 10.24	34.82 ± 10.46	** <0.001**
PH [*n* (%)]	13 (31.7)	28 (68.4)	** <0.001**

*Significant p results between ILFP (increased left ventricular filling pressure) and NLFP (normal left ventricular filling pressure) are reported by boldface. BMI, body mass index; BSA, body surface area; SBP, systolic blood pressure; DBP, diastolic blood pressure; LVMi, left ventricular mass indexed to body surface area; LVH, left ventricular hypertrophy; LAVi, left atrial volume indexed to body surface area; LAe, left atrial enlargement; E, E wave on transmitral Doppler; E/A, E wave on transmitral Doppler/A wave on transmitral Doppler; e' septal, septal tissue Doppler E wave; e' lateral, lateral tissue Doppler E wave; E/e' average, E wave on transmitral Doppler/mean tissue Doppler E wave; PAPm, mean pulmonary arterial pressure; TRv, tricuspidal regurgitation velocity; DLF, diastolic longitudinal force*.

Thirty-four patients showed normal left ventricular filling pressure (PCWP <15 mmHg, NLFP) and 33 patients showed increased left ventricular filling pressure (PCWP ≥ 15 mmHg, ILFP). No demographic features differed between the NLFP and ILFP group. NLFP group had lower LAVi (*p* < 0.001) and lower LAe rate (*p* = 0.010) than the ILFP one, while no differences were observed concerning LVMI and LVH (*p* = 0.092 and *p* = 0.967, respectively). EF and GLS were higher in NLFP, compared to ILFP (*p* = 0.012 and *p* = 0.031, respectively). Among left ventricular diastolic disfunction (LVDD) parameters, E/A, E/e' average, and e' septal differed between NLFP and ILFP (*p* < 0.050 for all), while e' lateral and tricuspidal regurgitation velocity (TRv) did not (*p* = 0.289 and *p* = 0.582, respectively). No gender-based differences were detected in the considered parameters.

According to current recommendation (6), 23 patients were classified as “normal filling pressure” (52.2 and 47.8% in NLFP and ILFP, respectively, *p* = 0.865), 20 patients as “increased filling pressure” (20.0 and 80.0% in NLFP and ILFP, respectively, *p* = 0.003), and 24 as “indeterminate filling pressure” (75.0 and 25.0% in NLFP and ILFP, respectively, *p* = 0.001).

### Hemodynamic Forces

As shown in Table 1, DLF differed between NLFP and ILFP groups (6.9 ± 3.6% vs. 5.2 ± 3%, *p* = 0.034), and at univariate regression analysis, it showed to be a possible predictor of PCWP class (Table 2).

**Table 2:** Univariate logistic regression and multivariate penalized regression.

	**Univariate logistic regression**			**Multivariate penalized regression**		
	**beta**	**OR [95% CI]**	***p*-value**	**Cutoff value**	**Beta**	**Score points**
**Demographic characteristics**						
Age (years)	0.02	1.02 [0.98–1.06]	0.239			
Sex (male)	−0.72	0.49 [0.18–1.33]	0.162			
Weight (kg)	−0.02	0.98 [0.95–1.01]	0.210			
Height (cm)	0.02	1.02 [0.97–1.07]	0.501			
BMI (kg/m^2^)	−0.10	0.91 [0.81–1.01]	0.070			
BSA (m^2^)	−0.96	0.38 [0.03–4.38]	0.441			
SBP (mmHg)	0.01	1.01 [0.99–1.03]	0.471			
DBP (mmHg)	0.00	1.00 [0.96–1.04]	0.869			
HR (bpm)	−0.03	0.97 [0.93–1.02]	0.238			
**Conventional echocardiographic parameters**						
EF (%)	−0.06	0.94 [0.90–0.98]	**0.003**	<40 %	1.22	**1**
GLS (%)	0.11	1.12 [1.03–1.22]	**0.012**			
LVMi (g/m^2^)	0.01	1.01 [1.00–1.02]	0.099			
RWT	−4.61	0.01 [0.00–0.74]	**0.049**			
LVH [*n* (%)]	−0.03	0.97 [0.21–4.44]	0.964			
LAVi (ml/m^2^)	0.08	1.09 [1.04–1.15]	** <0.001**			
LAe [*n* (%)]	1.60	4.98 [1.64–17.5]	**0.007**	34 ml/m^2^	0.82	**1**
E (cm/s)	2.10	8.18 [0.81–12.1]	0.096			
E/A	1.12	3.05 [1.39–6.71]	**0.006**			
e' septal (cm/s)	−0.28	0.75 [0.59–0.94]	**0.015**	<7 cm/s	0.61	**1**
e' lateral (cm/s)	−0.08	0.93 [0.81–1.07]	0.314			
E/e' average	0.16	1.17 [1.03–1.35]	**0.018**	>14	0.33	**0**
TRv (m/s)	−0.19	0.83 [0.40–1.61]	0.575			
EDVi (ml/m^2^)	0.04	1.04 [1.02–1.07]	**0.005**			
**Hemodynamic forces**						
DLF (%)	−0.18	0.84 [0.70–0.99]	**0.046**	<6.5 %	0.78	**1**

*Univariate logistic regressions are shown on the left side. Multivariate penalized regression, including variables that passed variable selection, is shown on the right side. Significant p results between ILFP and NLFP are reported by boldface. BMI, body mass index; BSA, body surface area; SBP, systolic blood pressure; DBP, diastolic blood pressure; LVMi, left ventricular mass indexed to body surface area; LVH, left ventricular hypertrophy; LAVi, left atrial volume indexed to body surface area; LAe, left atrial enlargement; E, E wave on transmitral Doppler; E/A, E wave on transmitral Doppler/A wave on transmitral Doppler; e' septal, septal tissue Doppler E wave; e' lateral, lateral tissue Doppler E wave; E/e' average, E wave on transmitral Doppler/mean tissue Doppler E wave; PAPm, mean pulmonary arterial pressure; TRv, tricuspidal regurgitation velocity; DLF, diastolic longitudinal force*.

**Figure 2:**
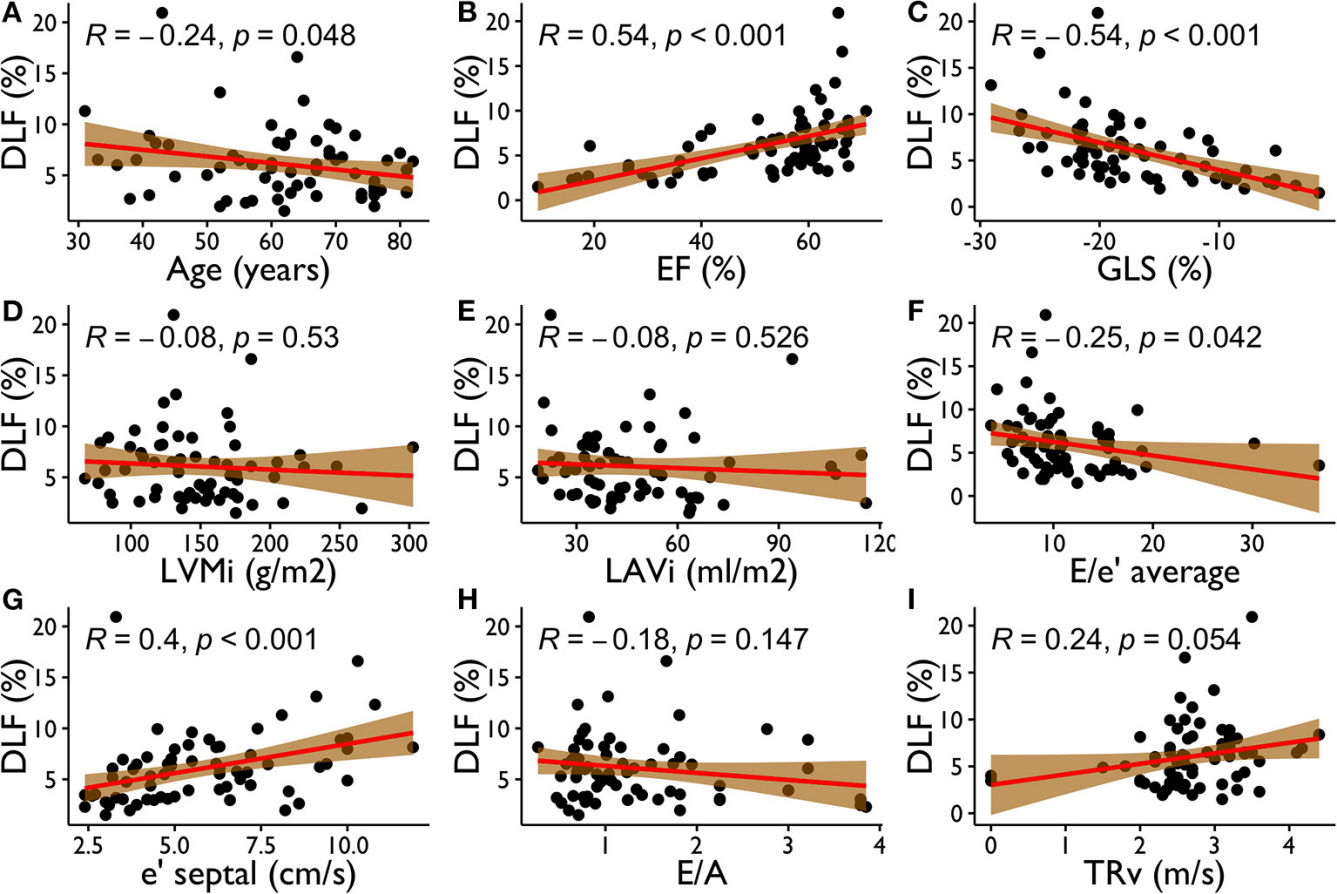
Correlations between DLF and Age (**A**), EF (**B**), GLS (**C**), LVMi (**D**), LAVi (**E**), E/e' average (**F**), e' septal (**G**), E/A (**H**), TRv (**I**). DLF, diastolic longitudinal force; EF, ejection fraction; GLS, global longitudinal strain; LVMi, left ventricular mass indexed to body surface area; LAVi, left atrial volume indexed to body surface area; e' septal, septal tissue Doppler E wave; E/e' average, E wave on transmitral Doppler/mean tissue Doppler E wave; E/A, E wave on transmitral Doppler/A wave on transmitral Doppler; TRv, tricuspidal regurgitation velocity.

Figure 2 reports scatter plots showing correlations between DLF and other variables, such as age, GLS, EF, and other conventional echocardiographic parameters for the assessment of LV filling pressure. DLF presented a moderate relationship with EF (*R* = 0.54, *p* < 0.001) and GLS (*R* = −0.54, *p* < 0.001). Weaker correlations were also present with age (*R* = 0.24, *p* = 0.048), E/e' average (*R* = −0.25, *p* = 0.008), and e' septal (*R* = 0.40, *p* < 0.001), while DLF did not become significantly associated to LVMi, LAVi, and TRv (*p* > 0.05 for all).

### PCWP Scoring System

As previously illustrated, 33 subjects (among 67 studied) presented ILFP. Univariate logistic regression analysis for prediction of ILFP is displayed in Table 2. In addition to the commonly known and recommended parameters for PCWP estimation (LAe, E/e' average, e' septal, TRv, and EF), only GLS, end-diastolic LV volume indexed to BSA, and DLF have proven to be predictive of ILFP. Lower DLF was associated with ILFP. Namely, we observed a 26% increase in risk for each DLF %-point less. Using Youden analysis, a cutoff of 6.5% proved to be the most accurate DLF threshold to identify ILFP.

**Figure 3:**
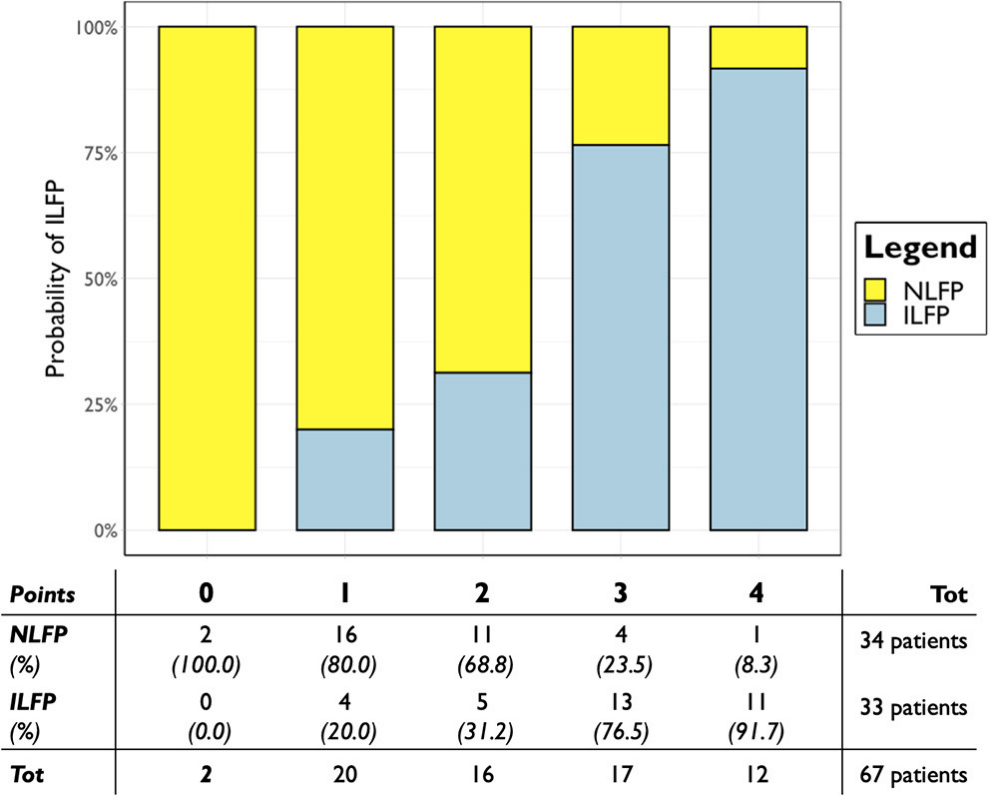
Predicted probability of PCWP > 15 mmHg according to the developed scoring system in the whole population. Scoring points are represented on the x-axis; probability of ILFP is represented on the y-axis. ILFP, increased left ventricular filling pressure; NLFP, normal left ventricular filling pressure; PCWP, postcapillary wedge pressure.

**Figure 4:**
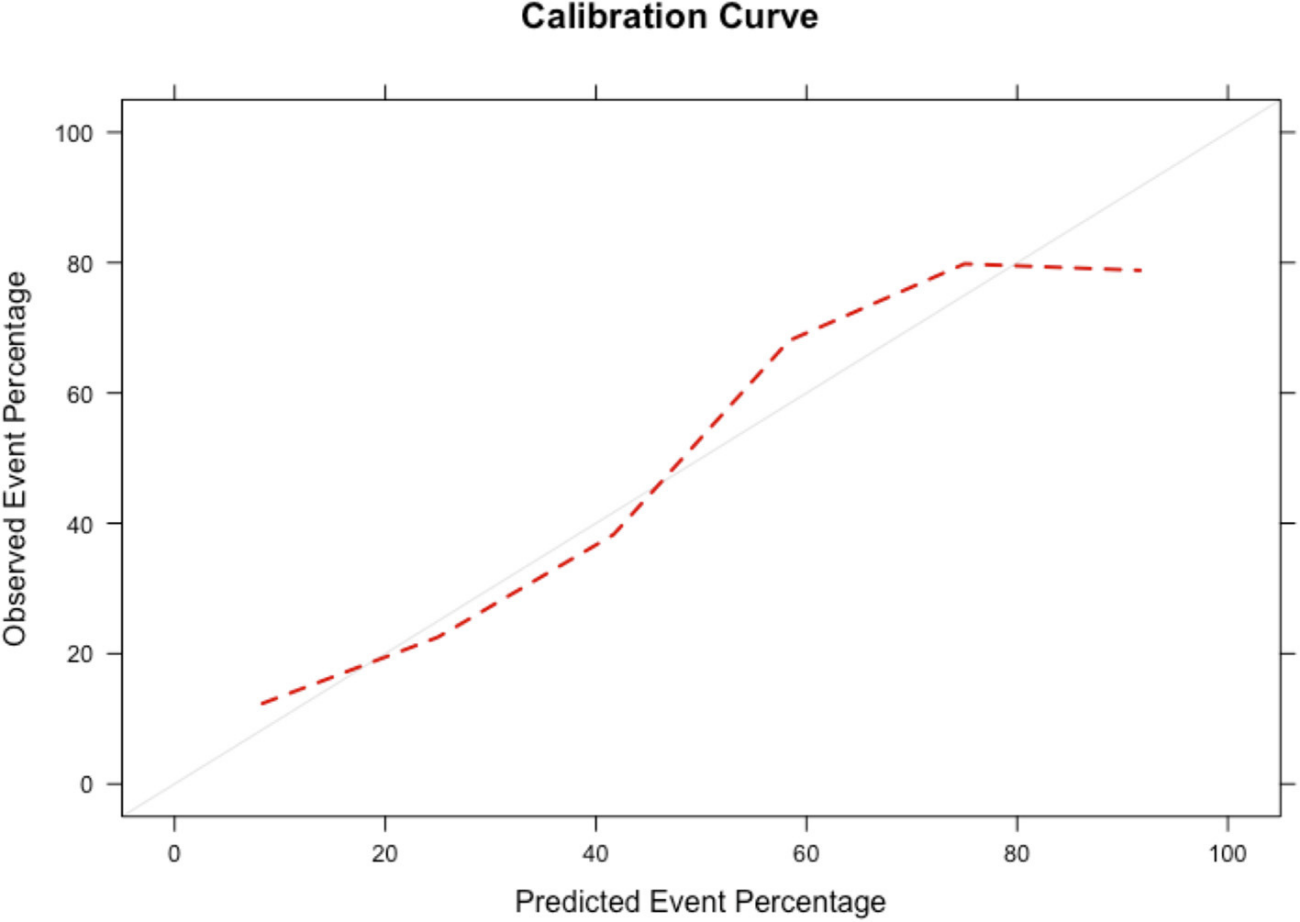
Smooth calibration plots for the validation of the scoring system by bootstrapping. The perfect condition is represented by the gray line, and the results of our calibration are represented by the red dashed line.

We performed variable selection by penalized regression (Supplementary Table 2) in order to develop a scoring system to predict LV filling pressure. DLF, EF, LAe, E/e' average, and e' septal have been inserted as categorical variables in the prediction model. The scoring points were weighted according to the β coefficients (Table 2). Figure 3 shows how the probability of ILFP rises with the increase in scoring. Internal validation was obtained through bootstrapping, showing optimal discrimination (Supplementary Figure 1) and calibration, with the smooth curve fitting the perfect condition (Figure 4, Supplementary Figure 2).

According to Youden analysis, the scoring system threshold has been set at two points, showing a sensitivity of 72.7% and a specificity of 85.3% for an overall AUC of 83% (*p* < 0.001). When DLF was not included within the scoring system, its sensitivity became 78.8% and specificity became 76.5%, for an overall AUC of 81% (*p* < 0.001). The developed score including DLF showed a positive predictive value of 82.8% and a negative predictive value of 76.3% in the study population, considering an ILFP prevalence of 49.3% (Supplementary Table 3).

When applied on patients classified as “indeterminate filling pressure” according to the current recommendation, the scoring system showed an accuracy of 87.5%, with 21 out of 24 patients correctly classified. Scoring values ≤ 2 correctly classified 17 out of 18 patients (94.4%) as NLFP, while scoring values ≥ 3 correctly classified four out of six patients (66.7%) as ILFP (Supplementary Table 3).

On the other hand, among patients who were not classified as “indeterminate,” our scoring system showed similar specificity to current recommendation (75 vs. 75%, *p* = 1.000) but a fairer sensitivity (74 vs. 59%, *p* = 0.157), although not statistically significant. Even NRI improved (0.15 [−0.15–0.45]; *p* = 0.329), although statistical significance was not reached.

**Figure 5:**
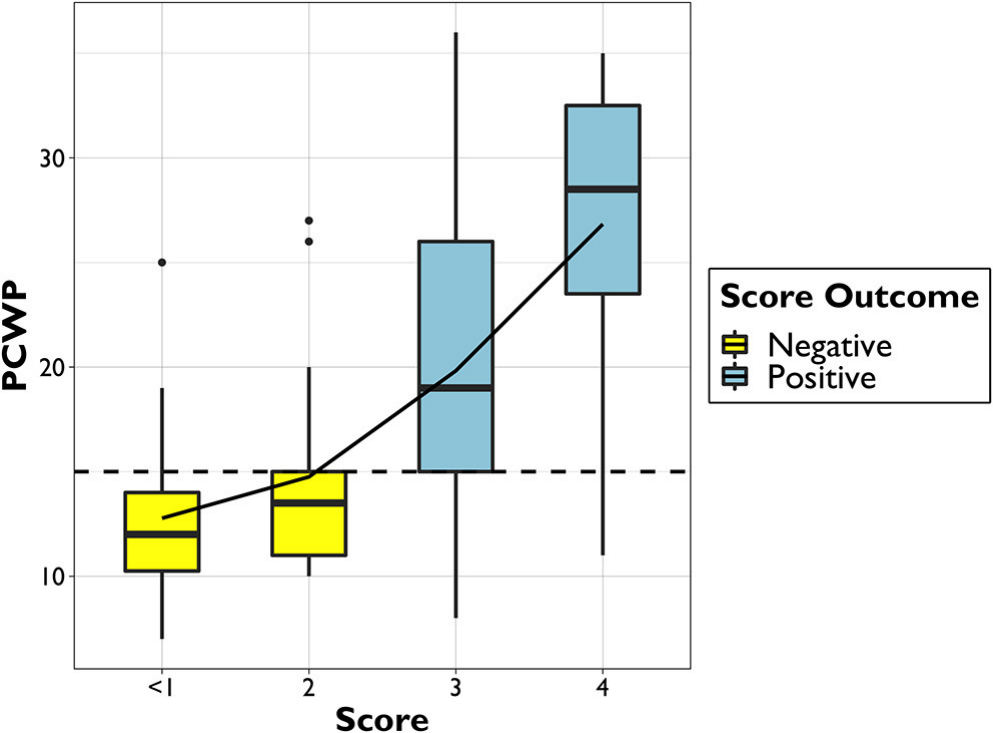
Boxplot of PCWP distribution among different scoring points. PCWP, postcapillary wedge pressure.

Moreover, the scoring system proved to be associated to the absolute values of PCWP (Figure 5) the median PCWP value was 12.0 [10.3–14.0] mmHg for patients with less than one point, 13.5 [11.0–15.0] mmHg for two points, 19.0 [15.0–26.0] mmHg for three points, and 28.5 [23.5–32.5] mmHg for four points.

## Discussion

This preliminary pilot study provides innovative data about blood flow analysis applied to the study of diastolic function. First, HDFs, DLF in particular, are associated with increased left ventricular filling pressure. Second, DLF can be included in a predictive LV filling pressure scoring system, contributing to identify patients with ILFP. Third, the developed scoring system was able to correctly classify the PCWP class of 21 out of 24 (87.5%) patients classified as “indeterminate filling pressure” by the current echocardiographic recommendation.

In the study population, morphometric characteristics were similar between NLFP and ILFP, reducing the related confounding risk in subsequent analyses. None of these variables were a plausible determinant of PCWP class.

Our study is a further confirmation of the well-known echocardiographic parameters associated with the diastolic function (LAe, e' septal, E/e' average, and E/A) included in the diagnostic flowchart suggested by the current guidelines (6). However, when applied to our population, the suggested diagnostic algorithm classified almost one-third of patients as “indeterminate filling pressure.”

Previous studies tried to develop simple methods, such as scores (26–28) and stepwise algorithms (29), to assess diastolic function, but due to results or complexity, these methods are not widespread in clinical practice. In this regard, Chubuchny et al. (28) developed a very promising algorithm that showed an excellent accuracy at internal validation, although it is limited by the large number of variables required and by the great influence attributed to mean pulmonary pressure.

Speckle-tracking analysis, particularly atrial strain, has already been applied to study the diastolic function. Left atrial strain has proven to be the most sensitive parameter in detecting diastolic dysfunction at an earlier stage, before it is evident through standard echocardiographic parameters (30). These data underline the importance of studying dynamic and functional characteristics, such as HDF, because they could highlight cardiac disorders before morphological parameters.

In a study aimed at assessing functional echocardiographic changes in patients with CRT (11), HDFs were superior even to strain analysis in identifying early abnormalities, proving to be an extremely promising approach. Nevertheless, to date, HDF analysis has never been studied in the context of LV diastolic function assessment and, to our knowledge, the present study is the first to perform this emerging technology for this purpose.

Among all measurable HDFs, we focused on DLF, which is closely related to the diastolic phase of the cardiac cycle, and for the first time, we proposed DLF as an index of the average force that is swapped along the longitudinal axis (apex to base) during diastole.

From the combination of classic echocardiographic variables and HDF analysis, we built a scoring system able to predict the presence of ILFP. The developed scoring system included EF, LAe, e' septal, and DLF. E/e' average did not reach statistical criteria to have one point assigned and, even forcing its presence in the final score, the overall accuracy did not improve (AUC: 0.83 vs. 0.82, *p* = 0.737). This finding seems to be in contrast with previous studies, describing E/e' average as a strong variable to distinguish precapillary from postcapillary pulmonary hypertension (26, 31). The reason for this discrepancy might be related to the presence of DLF within the model; this parameter has never been present before and could be a strong confounder to E/e' average. Anyway, in our population, E/e' average showed to be related to PCWP class, and we decided not to award any points to it in order to get a simpler score.

The developed score showed an optimal internal validation. Using two scoring points as diagnostic threshold to identify patients with a positive test, the predictive model reached high specificity (85.3%) and positive predictive value (82.8%) in detecting ILFP. The accuracy of the scoring system including HDF became higher by a few percentage points than the scoring system without DLF (83 vs. 81%, *p* = 0,580). Although statistical significance is not reached, we believe that these data are promising. Surely, in a larger population, the possibility to perform subgroup analyses and the stratification for systolic function would be required for a rigorous comparative approach.

Another clue to the possible value of our data is obtained by applying the scoring system to patients classified as “indeterminate filling pressure” by the current recommendations (6). In this subclass of subjects, our scoring system showed excellent specificity (94.4%) and negative predictive value (89.5%), proving to be a new potential tool to guide clinical decision. Among patients who were not classified as “indeterminate,” our scoring system showed to perform better than the current recommendation, although the low number of patients in this subset does not allow us to detect a statistically significant difference.

Furthermore, the scoring system showed a strong association with PCWP absolute value, even if not built for this purpose. It is therefore important to pay attention not only to the dichotomous outcome of the scoring system (more or less than two points), but also to its punctual value, as a severity index.

No clinical features (such as symptoms, clinical signs, or x-ray) were included in the present study for score development. These elements are mandatory in the heart failure diagnosis (1) and cannot be totally replaced by an echocardiographic scoring system, which should be considered additional to clinical data within a holistic diagnostic approach (4, 26). Finally, it must be emphasized that the aim of the study is not to question the current recommendations, but to focus attention on a new echocardiographic tool such as HDFs, which might be introduced in the assessment of diastolic function.

## Limitations

The present study is a retrospective study performed on patients who underwent RHC and echocardiography. The presented data are potentially very innovative, but the methodology of the study is exposed to some limitations.

First, the HDF analysis requires ultrasound images of discrete quality and a good ECG gating in order to perform a reliable speckle-tracking analysis. Since Right1 Study was not designed for this kind of investigation, the image quality was not always optimal for HDF assessment; a prospective analysis following good standards of speckle-tracking image acquisition (32) can certainly reduce this problem. This explains the high exclusion rate and therefore a possible selection bias. However, the comparison analysis between included and excluded subjects showed no significant differences.

Second, the actual knowledge and availability of HDFs prevent them from being applied in clinical practice, but our preliminary results bode well for future appropriately designed studies.

Third, a proper assessment of the HDF added value would require a larger study population, with subgroup analysis according to normal or reduced systolic function, even giving importance to the intra- and inter-operators' variability.

Moreover, since the scoring system has been obtained from a highly selected cohort of patients, its accuracy should be confirmed using an independent and prospectively acquired population. However, the good internal validation of the model seems to be promising. Finally, ILFP was defined on the basis of PCWP, as recommended (1), but LV end-diastolic pressures may also be used and sometimes considered a better gold standard (31).

## Conclusions

HDFs might be a novel echocardiographic parameter for the evaluation of diastolic function. DLF showed a great association with increased LV filling pressure. A new scoring system including DLF and other well-known echocardiographic variables showed a good accuracy in predicting PCWP class, both in the whole population and in patients classified as “indeterminate filling pressure” by the current recommendation. HDF analysis is a promising and still poorly explored domain of echocardiography. Further studies are needed in order to sharpen our knowledge on HDFs, allowing the evaluation of cardiac function from a new perspective.

## Data Availability Statement

The raw data supporting the conclusions of this article will be made available by the authors, without undue reservation.

## Ethics Statement

The studies involving human participants were reviewed and approved by Comitato Etico Interaziendale A.O.U. Città della Salute e della Scienza di Torino – A.O. Ordine Mauriziano. The patients/participants provided their written informed consent to participate in this study.

## Author Contributions

LA, FVa, FVe, and AM contributed to conception and design of the study. EA, AI, and CMa organized the database and performed echocardiography. PO and CMo performed right heart catheterization. LA, FVa, TF, and CG performed the statistical analysis. LA wrote the first draft of the manuscript. FVe, AA, GM, TF, and MC wrote sections of the manuscript. All authors contributed to manuscript revision, read, and approved the submitted version.

## Conflict of Interest

The authors declare that the research was conducted in the absence of any commercial or financial relationships that could be construed as a potential conflict of interest.

## Publisher's Note

All claims expressed in this article are solely those of the authors and do not necessarily represent those of their affiliated organizations, or those of the publisher, the editors and the reviewers. Any product that may be evaluated in this article, or claim that may be made by its manufacturer, is not guaranteed or endorsed by the publisher.

## Abbreviations

DLF: Diastolic Longitudinal Force
HDFs: hemodynamic forces
LV: left ventricle
ILFP: increased left ventricular filling pressure
LAVi: left atrial volume index
LAe: left atrial enlargement
LVH: left ventricular hypertrophy
LVMi: left ventricular mass index
NLFP: normal left ventricular filling pressure
PCWP: pulmonary capillary wedge pressure
RHC: right heart catheterization.
