# Supplementary material for: A Novel Approach to Left Ventricular Filling Pressure Assessment: The Role of Hemodynamic Forces Analysis
Source: Front Cardiovasc Med. 2021 Sep 8;8:704909. doi: 10.3389/fcvm.2021.704909 (PMC8455914; doi:10.3389/fcvm.2021.704909)
Supplement: Supplementary file 1 [file Data_Sheet_1.pdf]

SUPPLEMENTARY MATERIAL

Supplementary Figure 1 *– Discrimination Histogram*

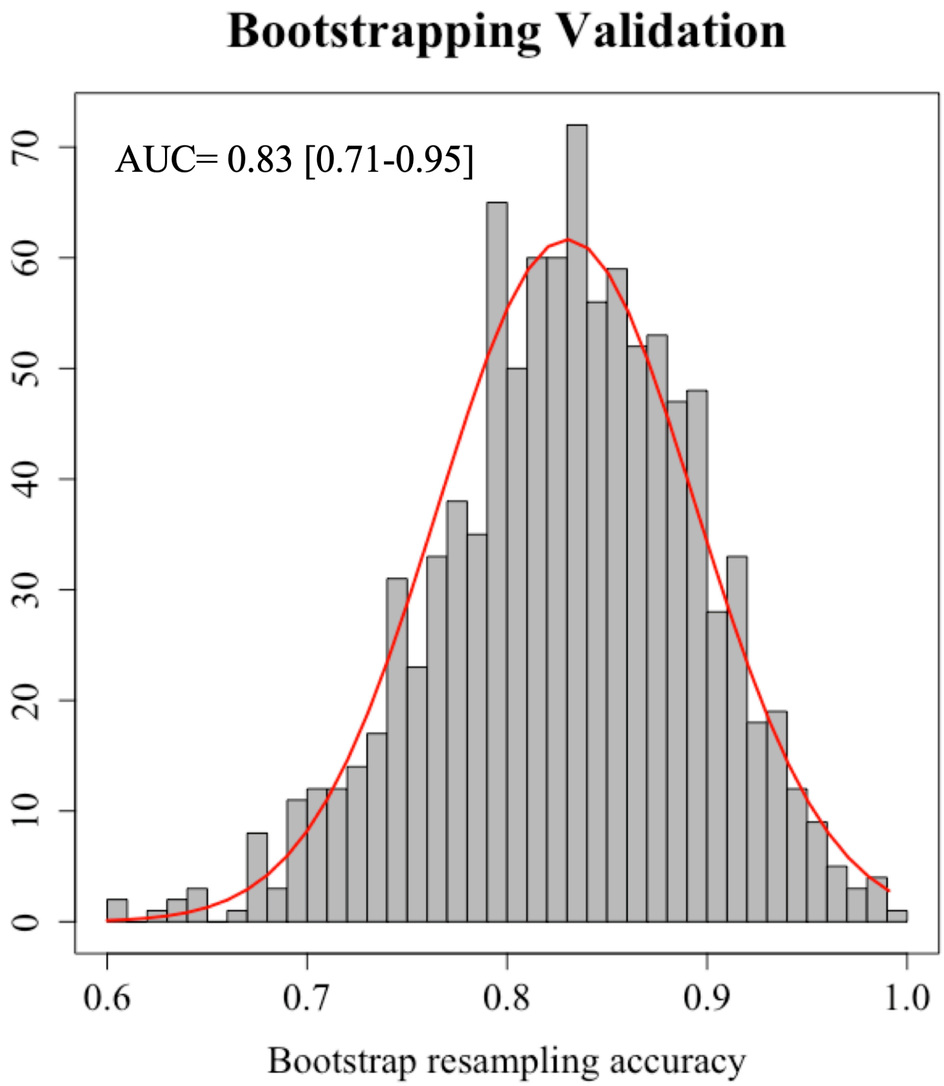

Distribution of bootstrapped AUC.

***Supplementary Figure 2** – Calibration Histogram*

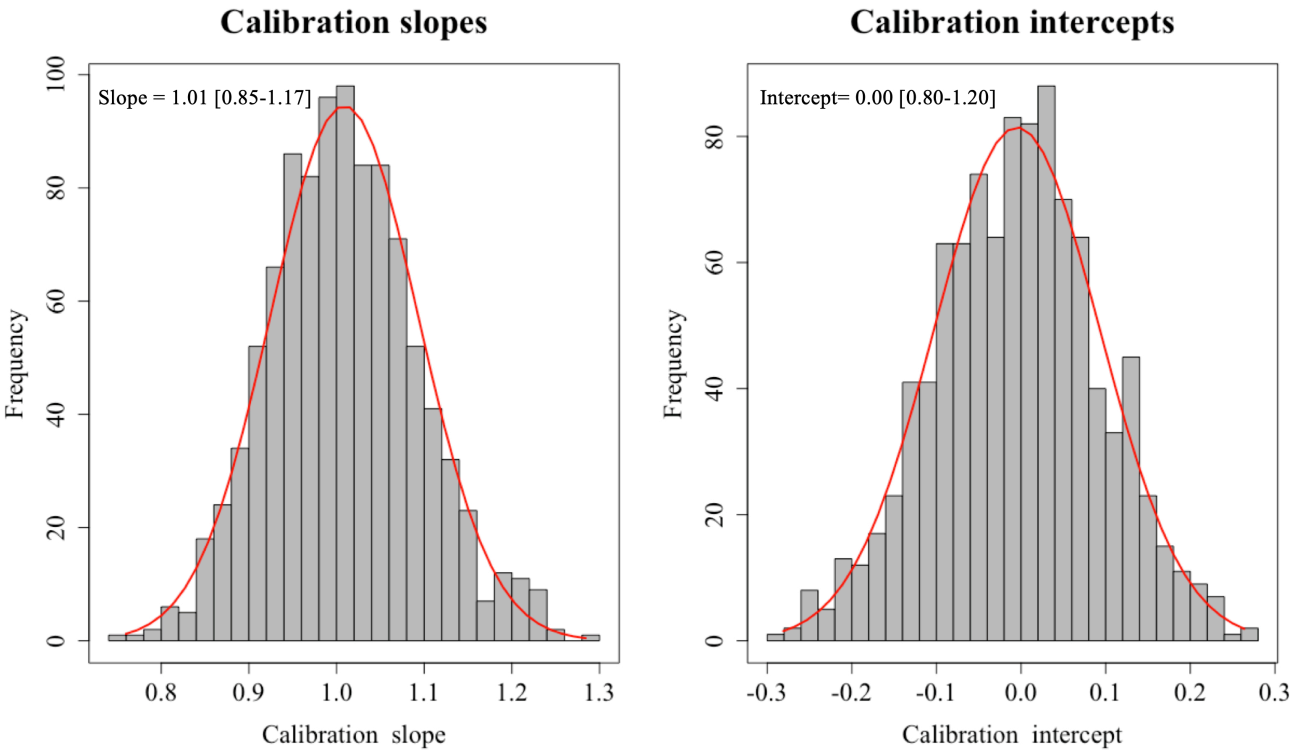

Distribution of bootstrapped slopes and intercept of calibration curve. The optimal is slope=1 and intercept=0.

Supplementary Table 1 – Comparison between included and excluded patients according to the presence of *HDFs analysis.*

	HDFs available		
	**No**<br>**N=81**	**Yes**<br>**N=67**	**p. value**
	**Demographic characteristics**		
**Age** (years)	58.27±14.19	62.2±13.1	0.084
**Sex** (Male) *(n(%))*	46 (55.4%)	41 (61.2%)	0.585
**Weight** *(Kg)*	73.8±14.8	71.8±14.7	0.417
**Height** *(cm)*	167±10.4	166±9.17	0.750
**BMI** (kg/m^2)	26.2 [23.0;29.6]	25.3 [22.8;28.5]	0.514
**BSA** *(m^2)*	1.82±0.21	1.79±0.20	0.442
**SBP** (mmHg)	128±20.2	132±20.0	0.383
**DBP** (mmHg)	73.9±13.3	73.5±12.3	0.905
	**Conventional Echocardiographic parameters**		
**LVMi** (g/m2)	101±32.2	149±45.1	**<0.001**
**RWT**	0.39 [0.33;0.47]	0.39 [0.32;0.44]	0.487
**LVH** (n(%))	33 (40.7%)	59 (88.1%)	**<0.001**
**LAVi** (ml/m^2)	40.8 [29.9;53.3]	41.4 [33.4;55.2]	0.621
**LAe** (n(%))	53 (65.4%)	46 (68.7%)	0.855
**TmE** (m/s)	0.63 [0.50;0.84]	0.69 [0.57;0.84]	0.323
**TmE/TmA**	1.00 [0.71;1.34]	0.99 [0.71;1.45]	0.782
**LtdiE** *(cm/s)*	7.80 [5.60;10.2]	8.30 [5.62;10.3]	0.967
**StdiE** *(cm/s)*	5.25 [3.77;7.00]	5.20 [3.82;7.20]	0.944
**TmE/MtdiE**	9.36 [7.13;13.8]	9.57 [7.77;14.0]	0.586
	**Right Heart Catheterization**		
**mPAP** *(mmHg)*	32.2±13.9	29.9±11.4	0.278
**PCWP** *(mmHg)*	14.00 [12.0;23.0]	14.0 [11.3;20.0]	0.667
**ILFP** *(n(%))*	40 (49.3%)	33 (49.3%)	1.000

Significant p results between ILFP and NLFP are reported by boldface. ***Legend:*** *BMI=body mass index; BSA=body surface area; SBP=systolic blood pressure; DBP=diastolic blood pressure; LVMi=left ventricular mass indexed to body surface area; LVH=left ventricular hypertrophy; LAVi=left atrial volume indexed to body surface area; LAe=left atrial enlargement; MAP=mean pulmonary arterial pressure; PCWP=postcapillary wedge pressure; ILFP=increased left ventricular filling pressure.*

Supplementary Table 2 – Scoring system development: *penalized logistic regression for variable selection.*

	beta
**Demographic characteristics**	
**Age** *(years)*	-
**Sex** *(male)*	-
**Conventional Echocardiographic parameters**	
**EF** *(%)*	-0.322
**GLS** *(%)*	-
**LVMi** *(g/m2)*	-
**LAVi** *(ml/m2)*	1.046
**TmE/MtdiE**	0.027
**StdiE** *(cm/s)*	-0.173
**TRv** *(m/s)*	-
**EDVi** *(ml/m2)*	-
**Hemodynamic Forces**	
**DLF** *(%)*	-0.058

Beta coefficients are available only for variables revealed to be strong predictors of PCWP class; other ones have been shrinkaged to zero. ***Legend:*** *LVMi=left ventricular mass indexed to body surface area; LAVi=left atrial volume indexed to body surface area; TmE/MtdiE=E wave on transmitral doppler/ mean tissue doppler E wave; StdiE=septal tissue doppler E wave; TRv=tricuspidal regurgitation velocity; DLF=diastolic longitudinal force.*

Supplementary Table 3 *– Performance of the scoring system with a 2-points threshold*

**Whole study population**	**NLFP** *n = 34*	**ILFP** *n = 33*	
Scoring points ≤ 2	29	9	*NPV = 76.3%*
Scoring points > 2	5	24	*PPV = 82.8%*
	Specificity = 85.3%	*Sensibility =72.7%*	*Accuracy = 79.1%*
**"Indeterminate filling pressure" according current recommendations**	**NLFP** *n = 18*	**ILFP** *n = 6*	
Scoring points ≤ 2	17	2	*NPV = 89.5%*
Scoring points > 2	1	4	*PPV = 80.0%*
	Specificity = 94.4%	*Sensibility = 66.7%*	*Accuracy = 87.5%*

Legend: *ILFP=increased left ventricular filling pressure; NLFP= normal left ventricular filling pressure, NPV: negative predictive value; PPV: positive predictive value.*
